# Enzymatic degradation is an effective means to reduce aflatoxin contamination in maize

**DOI:** 10.1186/s12896-021-00730-6

**Published:** 2021-12-17

**Authors:** Monica A. Schmidt, Yizhou Mao, Joseph Opoku, Hillary L. Mehl

**Affiliations:** 1 BIO5 Institute, University of Arizona, 1657 E. Helen St, Tucson, AZ 85718 USA; 2 Arid Land Agricultural Research Center, USDA Agricultural Research Service, 416 W Congress St, Tucson, AZ 85701 USA

**Keywords:** Maize, Aflatoxin, Enzyme, Degradation, Food security

## Abstract

**Background:**

Aflatoxins are carcinogenic compounds produced by certain species of *Aspergillus* fungi. The consumption of crops contaminated with this toxin cause serious detrimental health effects, including death, in both livestock and humans. As a consequence, both the detection and quantification of this toxin in food/feed items is tightly regulated with crops exceeding the allowed limits eliminated from food chains. Globally, this toxin causes massive agricultural and economic losses each year.

**Results:**

In this paper we investigate the feasibility of using an aflatoxin-degrading enzyme strategy to reduce/eliminate aflatoxin loads in developing maize kernels. We used an endoplasmic reticulum (ER) targeted sub-cellular compartmentalization stabilizing strategy to accumulate an aflatoxin-degrading enzyme isolated from the edible Honey mushroom *Armillariella tabescens* and expressed it in embryo tissue in developing maize kernels. Three transgenic maize lines that were determined to be expressing the aflatoxin-degrading enzyme both at the RNA and protein level, were challenged with the aflatoxin-producing strain *Aspergillus flavus* AF13 and shown to accumulate non-detectable levels of aflatoxin at 14-days post-infection and significantly reduced levels of aflatoxin at 30-days post-infection compared to nontransgenic control *Aspergillus*-challenged samples.

**Conclusions:**

The expression of an aflatoxin-degrading enzyme in developing maize kernels was shown to be an effective means to control aflatoxin in maize in pre-harvest conditions. This aflatoxin-degradation strategy could play a significant role in the enhancement of both US and global food security and sustainability.

**Supplementary Information:**

The online version contains supplementary material available at 10.1186/s12896-021-00730-6.

## Background

One quarter (25%) of the world’s crops are contaminated with mycotoxins [1]. Mycotoxins are toxic secondary compounds produced by a fungal source and can be responsible for massive agricultural losses world-wide. Aflatoxins, a class of mycotoxins, are produced by certain strains of *Aspergillus,* with two species, *A. parasiticus* and *A. flavus,* most frequently associated with agricultural losses [2, 3]. In the US, the major commodities that are susceptible to aflatoxins include maize, peanuts, cotton and tree nuts. Aflatoxins are toxic and carcinogenic to both animals and humans. If aflatoxin-contaminated food/feed is ingested it can result in hepatotoxicity, liver cancer, kwashiorkor and Reye’s syndrome [4–6]. Due to aflatoxins high toxicity, over 100 countries restrict the level in both food and feed [7], including the US [8]. Maize destined for humans and dairy cattle has the tightest limit, at 20 parts per billion (ppb) [9].

Maize is vital to both US agriculture and economy. The US provides over half of the maize global market [10]. In the US, field maize production is a $75B endeavor and comprises 95% of the total US grain production [11]. Worldwide there is an annual net loss of 16 million tons of maize due to aflatoxin contamination [12]. In the US alone, aflatoxin contamination of food/feed results in an estimated $52 M–$1.68B agricultural loss every year [5, 13]. Aflatoxin contamination in crops threatens agricultural development, food security and human health.

Current aflatoxin prevention mechanisms are inadequate. Breeding for fungal resistant crops [14], agronomic practices that minimize plant stress and thus reduce crop susceptibility to fungal infection and aflatoxin accumulation, pre-harvest biocontrol with non-aflatoxigenic *Aspergillus* strains [15] or *Trichoderma harzianum* strain [16, 17], improved storage methods post-harvest [18] and the utilization of trapping agents to block uptake of aflatoxins [19] are all currently used and still there are millions of tons of crops losses each year due to aflatoxin contamination. Biotechnology is a viable and necessary option to reduce incidence and severity of aflatoxin contamination in crops. Expression of various antifungal agents have shown varying degrees of success at the retardation of *Aspergillus* growth and a reduction in aflatoxin levels in transgenic plants [20–23]. Previous research demonstrated the RNAi suppression biotechnology method **h**ost-**i**nduced **g**ene **s**ilencing (HIGS) is a promising strategy to reduce aflatoxin produced from contaminating *Aspergillus* in pre-harvest conditions [23–27].

Another biotechnology strategy that might be as effective or used in parallel with RNAi suppression is biodegradation. This is based on the findings that some organisms are capable of degrading aflatoxins (for review [28]). Typically this methodology would involve the mixing of aflatoxin-contaminated items with the organism, or isolated enzymes, with degradation capacity and subsequent incubation. Screening revealed a number of organisms, such as soil bacteria, rumen bacteria, fungi and protozoa, which can degrade aflatoxin via an enzymatic reaction to non-toxic compounds [29–35]. Some aflatoxin-degrading organisms might not be ideal for use in food/feed items due to their own characteristics, but an aflatoxin-degrading enzyme has been characterized from the nontoxic and edible Honey mushroom, *Armillariella tabescens* [36].

In this study, we investigated the feasibility of degrading aflatoxin produced by a highly toxigenic *A. flavus* strain infecting maize by the expression of an aflatoxin-degrading enzyme in kernels utilizing an endoplasmic reticulum (ER) targeted sub-cellular compartmentalization stabilizing strategy. Transgenic kernels were characterized for transgene expression and infected with *A. flavus,* and subsequent aflatoxin concentrations in transgenic kernels were compared to concentrations in nontransgenic counterpart kernels.

## Results and discussion

### Embryo-specific expression of cassette

**Fig. 1:**
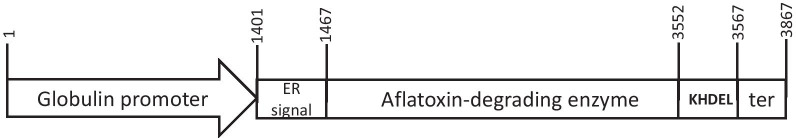
Schematic of the construct used to transform maize. A synthetic construct was manufactured consisting of 1.4 kb section of the embryo-specific *Zea mays* globulin 1 promoter driving expression of an ER-targeted plant codon optimized 2.166 kb open reading frame encoding for *Armillariella tabescenes’* aflatoxin-degrading enzyme. ER-targeting elements were added and consisted of a 5′ addition of a 22 amino-acid encoding signal sequence from the *Arabidopsis* chitinase gene and the 3′ addition of the ER retention KHDEL motif. Numbers are relative to indicate size of each construct element

An expression cassette was constructed where the 2166 bp open reading frame encoding for an aflatoxin-degrading enzyme previously isolated from Honey mushrooms *Armillariella tabescens* and characterized [36] was targeted to the ER by placing in-frame at the N-terminal a 22-amino acid ER signal sequence from the *Arabidopsis* chitinase gene and the nucleotides encoding for the known ER retention KHDEL sequence at the C-terminal of the protein. Both ER targeting sequences have been used successfully many times to localize transgenes to the ER in plant tissue [37–39] with the result being an enhanced accumulation of the ER-targeted protein. This aflatoxin-degrading enzyme protein sequence was searched via the World Health Organization (WHO) decision tree ranks to determine potential allergenicity and results deemed the protein to be very unlikely to be allergenic as it displays less than 1% homology to any known allergen (data not shown). The open reading from this fungal gene was codon-optimized for expression in maize seeds, ER-targeted and placed under an embryo-specific promoter (Fig. 1). The promoter was chosen by consideration of findings from analysis of maize cultivars that correlated levels of resistance to aflatoxin contamination was dependent on metabolic activity of the living embryo [40]. Likewise, aflatoxin-precursor metabolites were microscopically detected in embryo and aleurone tissue of maize kernels [41]. The color precursor to aflatoxin, NOR (norsolorinic acid), showed aflatoxin is specific to both embryo and aleurone maize kernel tissue 24 h post *Aspergillus* infection and then shifts to endosperm tissue in germinating maize kernels [41]. These findings indicated a transgenic aflatoxin-degrading strategy would be most efficient if expressed in embryo tissue. Of 500 screened embryo promoters from maize, the globulin-1 promoter was determined to have the strongest embryo-specific activity and was characterized as expression starting at late embryogenesis and throughout storage protein deposition [42]. For these reasons, a 1.4 kb region of the *Zea mays* globulin-1 promoter (Genbank accession AH001354.2) was synthetically manufactured and placed to direct expression of the ER-targeted codon optimized aflatoxin-degrading enzyme (Fig. 1).

### Aflatoxin-degrading enzyme expression in maize kernels

*Agrobacterium*-transformation was performed to obtain 10 independent bialaphos resistant putative transgenic maize lines containing the aflatoxin-degrading enzyme cassette. Initial screening of the embryo-specific aflatoxin-degrading enzyme transgenic maize plants was performed by genomic PCR to verify the inserted enzyme cassette was integrated into each transgenic line using primers specific to the embryo-expressed toxin-degrading enzyme cassette. All ten lines were confirmed genomic PCR positive and three transgenic lines were selected to be propagated and regenerated to the T_3_ stage and ensured stable transmission of the Enz transgene by repeated rounds of self-pollination and PCR screening of progeny each generation. 

**Fig. 2:**
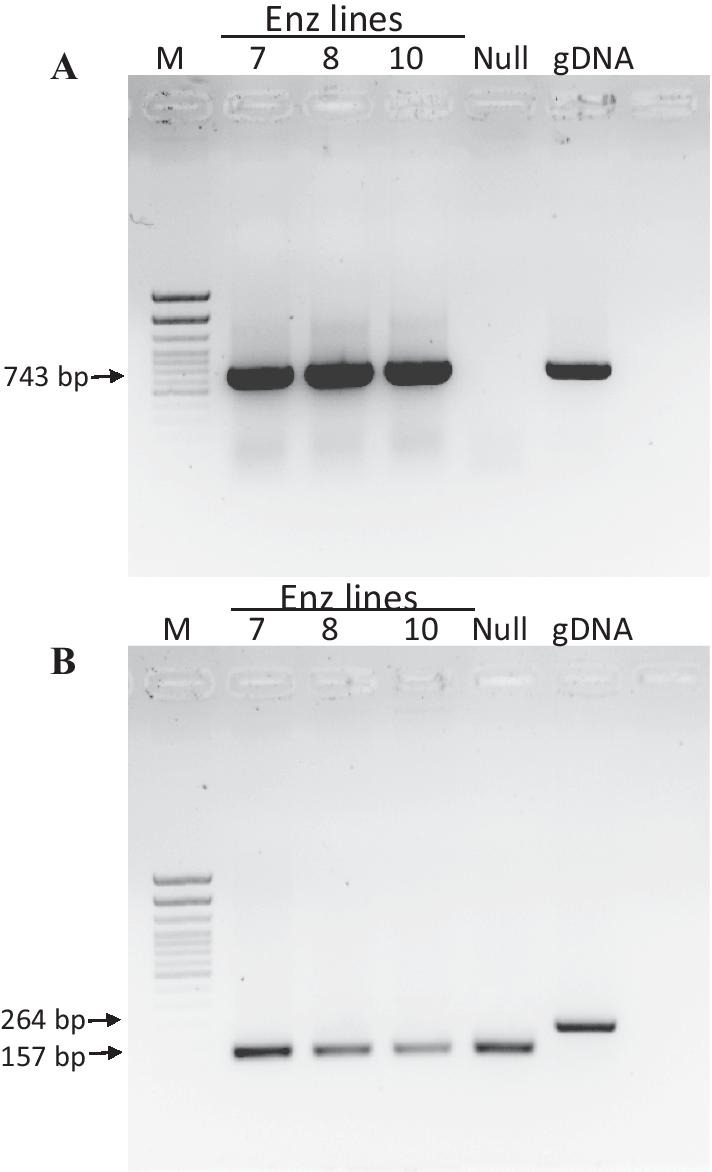
Expression of aflatoxin-degrading enzyme cassette in maize kernels. Expression of the Enz cassette was assessed using total RNA extracted from approximately 10 DAP developing kernels. **A** RT-PCR amplification using primers specific to the Enz open reading frame. The expected amplicon of 743 bp was detected in the 3 lines tested, Enz lines 7, 8, 10 and absent in a nontransgenic Null control. gDNA is genomic DNA isolated from transgenic Enz line 10. **B** RT-PCR amplification of a control endogenous gene actin using actin-specific primers that are adjacent to an intron. The expected cDNA 157 bp amplicon is detected in Enz transgenic lines 7, 8, 10 and a Null nontransgenic control. Genomic DNA (gDNA) amplicon actin expected size of 264 bp was not detected in the 3 Enz cDNA samples, indicating these samples do not contain any contaminating gDNA. M denotes DNA size marker

To investigate the expression of the inserted transgene cassette, total RNA was extracted from developing maize kernels and used to produce cDNA for reverse transcription PCR expression (RT-PCR) using primers specific to the inserted aflatoxin enzyme sequence and using an endogenous constitutive actin gene as a control. Figure 2 shows the expression results from developing kernels harvested from three independent Enz lines (Enz7, Enz8, and Enz10) compared to nontransgenic control (null) kernels. Expression of the Enz transgene was detected in all 3 lines analyzed as noted by the presence of the expected 743 bp amplicon (Fig. 2A). As the inserted Enz transgene does not contain an intron, in order to ensure cDNA was detected as opposed to residual genomic DNA, an endogenous gene that contains an intron was used as a control. Primers were designed to amplify the maize actin gene that would result in a 157 bp amplicon if cDNA was amplified, compared to a 264 bp amplicon when genomic DNA (gDNA) was amplified (Fig. 2B). The presence of only the 157 bp cDNA actin amplicon in all three Enz transgenic samples analyzed indicates the samples contain no genomic DNA and the Enz-specific PCR reactions were detecting the expression of the Enz transgene cassette in the developing kernels of the three lines (Enz7, Enz8 and Enz10).

**Fig. 3:**
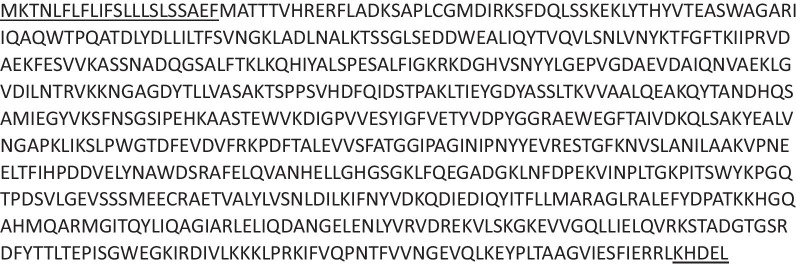
Detection of aflatoxin-degrading enzyme in maize kernel proteome by mass spectroscopy. Shown is the complete amino acid sequence of the inserted ER-targeted aflatoxin-degrading enzyme protein. Underlined is both the N-terminal ER signal peptide that is expected to be cleaved in the mature protein and the C-terminal ER-retention tag. Highlighted are the peptides detected in total soluble protein extracted from developing Enz maize kernels. This represents a 40% detection coverage and the inserted aflatoxin-degrading protein was detected in all three Enz transgenic lines assayed

To both confirm the presence of the inserted aflatoxin-degrading enzyme, mass spectroscopy analysis on developing transgenic maize kernels was performed. The three transgenic lines determined by RT-PCR to be expressing the inserted transcript (Fig. 2) were analyzed for total soluble protein as performed previously [37, 38, 43–46]. The decision to not add an epitope tag to the open reading frame of the toxin-degrading enzyme was made to ensure the correct folding and function of the enzyme. Developing maize kernels were harvested and flash frozen and total soluble protein was extracted and eventually digested with trypsin prior to mass spectrometry analysis. The resulting dataset was queried with the amino acid sequence of the Honey mushroom *Armillariella tabescens* aflatoxin-degrading enzyme (Genbank Accession AY941095) and exact peptide matches covering much of the sequence of the inserted protein were obtained (Fig. 3; Additional file 1: Table 1). The extensive coverage of the detected peptides of the inserted protein sequence and their presence in all three Enz transgenic developing protein samples with the absence of these peptides in the nontransgenic sample, indicates that the inserted aflatoxin-degrading enzyme is stably produced in developing maize kernels and it has accumulated to appreciable levels to be detected by mass spectroscopy. The subcellular targeting strategy used to express the aflatoxin-degrading enzyme likely enhanced the amount of this enzyme that accumulated in maize developing kernels, as this stabilization strategy has been shown to considerably elevate amounts of inserted proteins in maize kernels (for example [47, 48]) and other crop seeds [37, 39].

### Degradation is an effective means to reduce aflatoxin in maize kernels

**Fig. 4:**
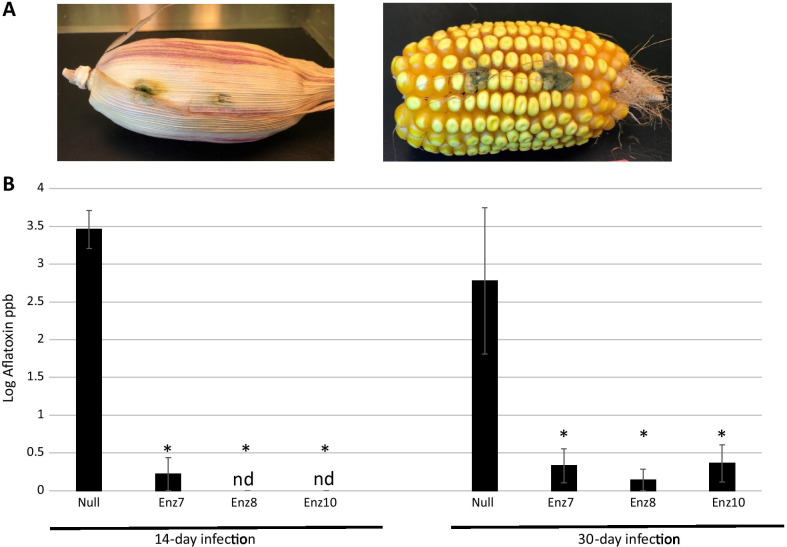
*Aspergillus flavus* infection and aflatoxin quantification in Enz transgenic maize. Freshly grown spore suspensions of *A. flavus* AF13 were injected into maize developing cobs and allowed to infect kernels. Shown **A** are two representative infection sites with husk intact (left) and husk removed (right) immediately prior to kernel harvest for aflatoxin quantification. Infected cobs were harvested at either 14- days or 30-days post-infection. Cobs had 4 infection sites each with up to 2 biological replicates for nulls and 4–5 biological replicates for each of the 3 transgenic lines (Enz7, Enz8, and Enz10). **B** Total aflatoxins were extracted from harvested kernels surrounding each infection site and quantified by thin layer chromatography followed by scanning densitometry. Shown for each sample is the average log ppb ± SE, nd denotes undetectable at a detection limit of 20 ppb. Averages of all three Enz transgenic lines were determined to be significantly different (denoted by *) from the nontransgenic null at both 14- and 30-day infection treatments as determined by student tests p < 0.05

The three transgenic Enz maize lines characterized for the insertion and expression of the aflatoxin-degrading enzyme encoding gene cassette were inoculated with *A. flavus* in pre-harvest conditions. At least three technical replicates for the three stable biological transgenic Enz lines were used in the challenges along with nontransgenic (null) counterpart controls. Multiple infection sites were made into developing cobs of biological replicate plants of the 3 Enz transgenic lines (Enz7, Enz8 and Enz10) along with side-by-side greenhouse grown nontransgenic null plants by inoculating 8–10 days after pollination (DAP) cobs with 10 μl of a freshly grown *A. flavus* AF13 spore suspension (1.0 × 10^7^ spores/ml) in sterile distilled water (Fig. 4A). The infections were allowed to progress for 14 or 30 days duration. After infections, all live kernels surrounding each infection site were harvested as described previously [25] and total aflatoxins were extracted, separated by thin layer chromatography (TLC), and quantified on TLC plates using scanning densitometry. Figure 4B shows aflatoxin concentrations after developing kernels were infected with *A. flavus* and the infection was allowed to occur for 14 days. Compared to null nontransgenic maize developing kernels, the three aflatoxin-degrading enzyme expressing transgenic lines (Enz7, Enz8 and Enz10) had significantly reduced aflatoxin concentrations (student t-test p < 0.05) with 2 of the lines having undetectable levels of aflatoxin (< 20 ppb). As shown in Fig. 4B, after a 14-infection day duration, nontransgenic null maize kernels contained 3.46 ± 0.25 ppb log aflatoxin compared to 0.22 ± 0.22 ppb in transgenic line Enz 7 and non-detectable levels in both Enz8 and Enz10 transgenic lines. The TLC methodology employed has a detection limit of 20 ppb (log value 1.30 ppb) and given that 20 ppb is the tightest aflatoxin limit in the US for food items destined for direct human consumption, this aflatoxin-degrading enzyme method of the reduction or elimination of this carcinogenic compound from maize, or similarly *Aspergillus*-infected crops, is very feasible as this strategy should play a significant role towards eliminating crop losses due to this fungal contaminant.

Likewise, Fig. 4B shows aflatoxin loads from similarly infected developing kernels of transgenic Enz maize where the *A. flavus* infection was allowed to proceed for 1 month before harvest and aflatoxin quantification. Again, as with the 14-day infection period, all 3 expressing aflatoxin-degrading enzyme transgenic maize lines displayed significantly reduced aflatoxin loads compared to the nontransgenic null kernels. Although there was substantial variation, the transgenic lines accumulated at least a 90-fold reduction in aflatoxin after a 30-day *A. flavus* infection period with null kernels having an average 2.78 ± 0.97 ppb log aflatoxin with Enz 7 accumulating 0.33 ± 0.22 ppb, Enz 8 accumulating 0.14 ± 0.14 ppb and Enz 10 accumulating 0.36 ± 0.29 ppb. Variation in aflatoxin concentration in *Aspergillus*-infected maize has been previously reported to increase as the aflatoxin concentration increases [49]. All aflatoxin accumulated in the three Enz transgenic lines was determined to be significantly reduced from the nontransgenic null controls by student tests p < 0.05. Even with a 30-day infection period, the embryo-expressed aflatoxin-degrading enzyme was able to convert the carcinogenic aflatoxin produced by the contaminating *A. flavus* fungus to substantially reduced levels in all three transgenic maize lines.

As a refinement of this biotechnology approach, perhaps the aflatoxin-degrading enzyme could be expressed in the edible portion of maize but also placed under a pathogen-induced promoter. Of the pathogen responsive (PR) genes studied, fungus-infection inducible expression of maize promoters have been reported. The *ZmPR4* gene encoding a class II chitinase of the PR-4 family of PR proteins, the *mpi* gene encoding a proteinase inhibitor, and the *PRms* gene encoding a member of the PR-1 family of PR proteins were characterized and determined that when driving the expression of an antifungal protein in rice, *ZmPR4* conferred the highest level of resistance [50]. In future reiterations of this degradation work, instead of using an embryo-specific promoter as was used in this study, a fungal-infection responsive promoter might be used to drive the aflatoxin-degrading enzyme production so the degradation of the aflatoxin produced can keep pace with the production of the toxin while the *Aspergillus* infection progresses. In this study, the trend is that the degradation capacity dwindles over time in comparison of the undetectable levels of aflatoxin seen in 14-day challenged kernels to the small, yet detectable, aflatoxin levels accumulating in the 30-day challenged kernels (Fig. 4B). This indicates the degradation strategy losses some of its effectiveness as the *Aspergillus* infection continues, likely due to the limited embryo-driven expression of the enzyme being able to convert the aflatoxin produced by an increasing growing mass of *Aspergillus*. The enzyme-degradation strategy’s long-term effectiveness might be enhanced by using a fungal-infection induced promoter system to drive its expression in the edible portion of crops that are susceptible to *Aspergillus*-infection and aflatoxin accumulation. Additionally, this degradation biotechnology strategy could be used in parallel with other successful aflatoxin suppression strategies, such as HIGS [25] or antifungal growth technologies [20–23] to retard the initial *Aspergillus* infection and subsequent growth.

## Conclusions

Aflatoxin contamination is responsible for substantial economic losses worldwide in addition to being the causal agent of significant deleterious health effects. With the already staggering economic losses in the US due to aflatoxin in maize, losses are likely to increase in the future due to forecasted elevated temperatures and drought stress—as both of these factors have been shown to increase the infection of *Aspergillus* on maize and the level of aflatoxin produced [51] (for review). In this paper, we demonstrate proof-of-concept that a biotechnology approach using the expression of an enzyme capable of degrading aflatoxins in the edible portion of maize is an effective means to eliminate/minimize this carcinogenic compound from food/feed chains. This enzyme-degradation strategy can be added to the arsenal of approaches used to combat this agricultural and health hazard compound and could play a substantial role in alleviating the current situation where an estimated 1 in every 3 humans suffer from food insecurity and/or nutrient deficiency [52].

## Methods

### Aflatoxin-degradation expression cassette

The aflatoxin-degrading enzyme from the Honey fungus *Armillariella tabescens* (GenbankAccession AY941095) consisting of a 2166 bp open-reading frame with both ER-signal andER-retention tags flanking the aflatoxin-degrading encoding a 695 amino acid protein was synthesized (Celtek Genes) using a plant codon optimization table. This enzyme’s open reading frame was placed in-frame between elements to subcellularly localize the protein to the ER by the addition of the 22 amino-acid ER signal sequence from the *Arabidopsis* chitinase gene at the 5′ end and the ER retention KHDEL motif at the 3′ end of the open reading frame as previously described [37–39]. The ER-targeted enzyme-encoding gene was then placed under the direction of an embryo-specific promoter. A 1.4 kb region of the *Zea mays* globulin-1 promoter (Genbank Accession AH001354.2) was synthetically manufactured and used in the expression cassette for the embryo-specific expression of an aflatoxin-degrading enzyme in maize. The embryo-directed expression of the aflatoxin-degrading enzyme cassette was subsequently cloned into an *Agrobacterium tumefaciens* plasmid pTF1010.1 that contains the constitutively expressed selectable marker *bar* resistance gene (phosphinothricin acetyltransferase). The resultant cassette was hereafter referred to as glob::Enz.

### Transgenic maize production

Transgenic maize *(Zea mays* Hi II hybrid A 188 and B73 background) expressing the glob::Enz cassette were produced by the Iowa State University Plant Transformation Facility (www.biotech.iastate.edu) using *Agrobacterium*-mediated transformation protocol [53]. Initial plant material was provided by the facility. Plantlets from ten putative transgenic lines were obtained after tissue-culture selection on media containing the selectable agent, bialophos. Each transgenic line was confirmed by PCR to be containing the glob::Enz cassette by genomic PCR using primers specific to the cassette (Enz-For 5′-GTTGGCAGATCTTAACGCTCT-3′, Enz-Rev 5′-CTTCCCATTCAGCCCTACCTC-3′ producing an expected amplicon of 743 bp). Standard PCR conditions were used in the PCR screening: 50 ng genomic DNA, 1X Taq DNA polymerase buffer, 2U Taq polymerase (New England Biolabs), 250 μM of each dNTP and 200 nM of each primer with PCR conditions of an initial denaturation (94 °C, 4 min) and 45 amplification cycles (94 °C, 30 s; 55 °C, 30 s; 72 °C 60 s) followed by a final elongation step (72 °C, 7 m). Transgenic lines were grown to the T_3_ generation by repetitive self-pollination and ensured stable transmission of the Enz cassette by performing Enz-specific PCR reaction screening on progeny each generation.

### Transgene expression in transgenic maize kernels

Transgenic glob::Enz transgenic maize lines were screened by expression of the inserted cassette of interest by detecting the Enz transcript in developing kernels. RNA was extracted using RNeasy Kit (Qiagen) by grinding approximately 10 DAP kernels in liquid nitrogen harvested from three stable transgenic lines (Enz-7, Enz-8, Enz-10) and nontransgenic (null) control kernels. First-stand cDNA synthesis was performed using 1 μg of total RNA per sample, 9 μl of 2 M betaine monohydrate (Sigma) and random primers using RevertAid First Strand cDNA Synthesis Kit (Thermo Scientific) according to the manufacturer’s instructions. Standard PCR conditions were as described in transgenic maize screening above using primers specific to the Enz open reading frame (as above) and primers specific to the control maize actin gene (Phytozome v12.1 database ID GRMZM2G126010; Actin-For 5′-CCCTCTCAACCCCAAGGC-3′, Actin-Rev 5′-GCTCACACCATCACCGGAA-3′). The maize actin primers were designed adjacent to an intron, so there was an expected amplicon size differential if genomic DNA or cDNA was amplified, 264 bp and 157 bp, respectively. PCR amplicon products were separated on a 1% (w/v) agarose gel (Sigma-Aldrich) mixed with ethidium bromide (0.5 μg/ml) (Sigma-Aldrich) using a 100 pb DNA Ladder (Fisher Scientific) and subsequently imaged under ultraviolet light.

### Protein preparation and data acquisition

Total protein was isolated from developing maize kernels according to a modified phenol method [54, 55]. The proteomics work was done at the Proteomics and Mass Spectrometry Core, ICBR, University of Florida. Proteins were dissolved in protein buffer (8 M Urea, 0.1% SDS, 25 mM Triethylammonium bicarbonate, pH 8.0) and quantified following a previous method [55, 56]. Protein assays were performed to quantify purified proteins by the EZQ™ Protein Quantification Kit (Thermo Fisher Scientific, San Jose, CA, USA) with the SoftMax Pro Software v5.3 under the SpectraMax M5 (Molecular Devices, LLC). For each sample, a total of 30 μg of protein were reduced with 40 mM DTT, alkylated with 100 mM of iodoacetamide, and trypsin-digested (at an enzyme to protein ratio (w/w) of 1:100). Tryptic digested peptides were desalted with C18-solid phase extraction (The Nest Group, INC, Southborough, MA). An Orbitrap Fusion Tribrid Mass Spectrometer system (Thermo Fisher Scientific, San Jose, CA, USA) was used with collision ion dissociation (CID) in each MS and MS/MS cycle. The MS system was interfaced with an ultra-performance Easy-nLC 1200 system (Thermo Fisher Scientific, Bremen, Germany). A total of 2 μg of each sample was loaded onto a Acclaim Pepmap 100 pre-column (20 mm × 75 μm; 3 μm-C18) and then separated on a PepMap RSLC analytical column (500 mm × 75 μm; 2 μm-C18) at a flow rate of 250 nl/min of solvent A (0.1% formic acid, 99.9% water (v/v)), followed by a linear increase from 2 to 35% solvent B (0.1% formic acid, 80% acetonitrile, 19.9% water (v/v)) in 160 min and from 35 to 80% solvent B in 5 min, then ramping up to 98% solvent B in 1 min, and stayed for 14 min.

### Data dependent decision tree acquisition

The mass spectrometer was operated in MS/MS mode scanning from 350 to 2000 m/z. The maximum ion injection times for the survey scan and the MS/MS scans were 35 ms. MS1 spectra were recorded at resolution at 120,000 FWHM from 350–2000 m/z with quadrupole isolation was followed by one MS/MS scans of the most intense precursor ions in the linear ion trap. The automated gain control (AGC) target was set to 2 × 105, with a maximum injection time of 50 ms. The quadrupole was used for precursor isolation with an isolation window of 1.3 m/z. Only precursors with charge states 2–8 with an intensity higher than 1 × 104 were selected for fragmentation. The monoisotopic precursor selection (MIPS) filter was activated. The option to inject ions for all available parallelizable time was selected. Targeted MS2 spectra with different fragmentation parameters were acquired (Additional file 1: Table 1) and were performed in the ion trap with CID fragmentation (Rapid; NCE 35%; maximum injection time 35 ms; AGC 1 × 104). The normalized collision energy (NCE) was set to 35% for each fragmentation method and one microscan was acquired for each spectrum.

### Proteomics data search and analysis

Tandem mass spectra were extracted by Proteome Discoverer version 2.5. All MS/MS samples were analyzed were processed by a thorough database searching considering biological modification and amino acid substitution against an Uniprot non-redundant maize database (99,207 entries download on September 10, 2021) with decoy option using MASCOT 2.7.01 (Matrix Science Inc., Boston, MA, USA) with the following parameters: peptide tolerance at 10 ppm, tandem MS tolerance at ± 1.00 Da, peptide charge from 2+ to 6+, trypsin as the enzyme, Carbamidomethyl (C) as fixed modifications, and oxidation (M) and phosphorylation (S, T, Y) as variable modifications. The false discovery rate (FDRs) of proteins was controlled under 5%. Scaffold (version Scaffold_4.2.1, Proteome Software Inc., Portland, OR) was used to validate MS/MS based peptide and protein identifications. Peptide identifications were accepted if they could be established at greater than 99.9% probability by the Peptide Prophet algorithm [41] with Scaffold delta-mass correction. Protein identifications were accepted if they could be established at greater than 95.0% probability. Protein probabilities were assigned by the Protein Prophet algorithm [57]. Proteins that contained similar peptides and could not be differentiated based on MS/MS analysis alone were grouped to satisfy the principles of parsimony. Proteins sharing significant peptide evidence were grouped into clusters. The spectral count for each protein was calculated by assigned a peptide from that protein with high confidence.

### *Aspergillus flavus* culture propagation

*Aspergillus flavus* isolate AF13 [58] from the USDA-ARS Aflatoxin Biocontrol Lab culture collection was grown from long-term silica gel stocks by placing a single silica granule on the center of 5/2 agar (5% V-8 vegetable juice and 2% agar, pH 5.2) and incubating the plate in the dark at 31 °C for 5 days. Agar plugs (7–10 per vial) were transferred to water vials which containing 3.5 ml of ddH_2_O. Spore suspensions (15 µl) from water vial stocks were seeded in the center well of 5/2 agar plates, and after incubation at 31 °C for 5–7 days, spores were picked up from plates using sterile cotton swabs and suspended in 10 ml of sterile 0.02% Tween-80. Spore suspensions were vortexed, and 1.2 ml of the suspension was added to 10.8 ml of 50% ethanol. Turbidity in nephelometric turbidity units (NTUs) was measured using a calibrated turbidimeter (Orbeco-Hellige Farmingdale NY, model 965–10). The final spore concentration was calculated using a standard curve for NTU versus spores/ml using the formula: spores/ml = NTU × 49,937. The spore suspension was then diluted to a final concentration of 1.0 × 10^7^ spores/ml in sterile distilled water.

### *Aspergillus flavus* infection assays and aflatoxin quantification

At 8 to 10 DAP, ears on transgenic maize plants and nontransgenic null control plants grown side-by-side under greenhouse conditions were wounded at four spots by pushing a 3-mm diameter cork-borer through the husk to a depth of approximately 5 mm. Each wound was inoculated with 10 μl of the *A. flavus* conidial suspension. In each experiment, 3 to 5 ears of each transgenic line (Enz 7, Enz 8, and Enz 10) and at least one non-transgenic null ear were inoculated. After 30 and 14 days in the first and second experiment, respectively, ears were harvested and dried at 45 °C for 3 to 4 days. Eight to nine kernels surrounding each inoculated wound were removed from the ears, weighed, and ground. For each sample, total aflatoxins (aflatoxin B_1_ + aflatoxin B_2_) were extracted from 1.5 g ground kernels with 15 ml of 70% methanol, and extracts were separated using thin layer chromatography (TLC) and aflatoxin was quantified using scanning densitometry as described previously [59]. Briefly, 12 μl of extract was spotted on 20 × 20–cm TLC glass plates (Silica Gel 60 F254, Millipore) along with an aflatoxin standard (Aflatoxin Mix Kit-M, Supelco, Bellefonte, PA) and plates were developed with diethyl ether:methanol:water (96:3:1). The presence or absence of aflatoxins B_1_ and AB_2_ were confirmed visually under ultraviolet light (365 nm) and quantified on plates using scanning fluorescence densitometry with a CAMAG TLC Scanner 3 (Camag Scientific Inc.). Quantities of aflatoxin relative to the standard were used to calculate total ng aflatoxin per g kernels (parts per billion; ppb). Values are presented as average log ppb ± standard error and determined to be significant at p < 0.05 by performing student t-tests comparing each transgenic event to the nontransgenic control.

## Supplementary Information

**Additional file 1.** **Supplementary Table 1.** Peptides generated from mass spectroscopy analysis of the honey mushroom aflatoxin-degrading enzyme.

## Data Availability

All data generated by this study are included in this article. No data was generated that needed deposition into databases. Material is available from the corresponding author on reasonable request.

## Abbreviations

ER: Endoplasmic reticulum
DAP: Days after pollination
TLC: Thin layer chromatography
ppb: Parts per billion
TEAB: Tetraethylammonium bromide
